# Unfertilized frog eggs die by apoptosis following meiotic exit

**DOI:** 10.1186/1471-2121-12-56

**Published:** 2011-12-23

**Authors:** Alexander A Tokmakov, Sho Iguchi, Tetsushi Iwasaki, Yasuo Fukami

**Affiliations:** 1 Research Center for Environmental Genomics, Kobe University, Rokko dai 1-1, Nada, Kobe 657-8501, Japan; 2 Graduate School of Science, Kobe University, Rokko dai 1-1, Nada, Kobe 657-8501, Japan

## Abstract

**Background:**

A characteristic feature of frog reproduction is external fertilization accomplished outside the female's body. Mature fertilization-competent frog eggs are arrested at the meiotic metaphase II with high activity of the key meiotic regulators, maturation promoting factor (MPF) and cytostatic factor (CSF), awaiting fertilization. If the eggs are not fertilized within several hours of ovulation, they deteriorate and ultimately die by as yet unknown mechanism.

**Results:**

Here, we report that the vast majority of naturally laid unfertilized eggs of the African clawed frog *Xenopus laevis *spontaneously exit metaphase arrest under various environmental conditions and degrade by a well-defined apoptotic process within 48 hours after ovulation. The main features of this process include cytochrome *c *release, caspase activation, ATP depletion, increase of ADP/ATP ratio, apoptotic nuclear morphology, progressive intracellular acidification, and egg swelling. Meiotic exit seems to be a prerequisite for execution of the apoptotic program, since (i) it precedes apoptosis, (ii) apoptotic events cannot be observed in the eggs maintaining high activity of MPF and CSF, and (iii) apoptosis in unfertilized frog eggs is accelerated upon early meiotic exit. The apoptotic features cannot be observed in the immature prophase-arrested oocytes, however, the maturation-inducing hormone progesterone renders oocytes susceptible to apoptosis.

**Conclusions:**

The study reveals that naturally laid intact frog eggs die by apoptosis if they are not fertilized. A maternal apoptotic program is evoked in frog oocytes upon maturation and executed after meiotic exit in unfertilized eggs. The meiotic exit is required for execution of the apoptotic program in eggs. The emerging anti-apoptotic role of meiotic metaphase arrest needs further investigation.

## Background

The African clawed frog, *Xenopus laevis*, is an important model organism in developmental biology. *Xenopus *oocytes, eggs and early embryos have been widely used in cell cycle studies, which provided a basis for the current understanding of meiotic and mitotic transition. Most control mechanisms that operate in maturing oocytes, fertilized eggs, and early embryos have been first established in *Xenopus laevis *[reviewed in refs. [1,2]]. However, the fate of unfertilized eggs in this species has received little attention.

Fully grown *Xenopus *oocytes of the stage VI are naturally arrested in the prophase of the first meiotic division with the intact nuclear envelope and partially decondensed chromatin. Immature fully grown *Xenopus *oocytes are not competent to fertilization and can be arrested at this stage in the ovaries for many months. During ovulation, the steroid hormone progesterone, secreted from surrounding follicle cells, induces oocyte transition from prophase I to metaphase II in the process of meiotic maturation. In frogs, the term "egg" is conventionally used for the ovulated female gamete arrested in the metaphase of the second meiotic division. High activities of the key meiotic regulators, maturation promoting factor (MPF, a complex of cyclin B and Cdk1 kinase) and cytostatic factor (CSF, which includes activated MAPK pathway) have been established to maintain metaphase II arrest in mature *Xenopus *eggs [3-6]. The meiotic arrest is essential for the embryonic development as it allows mature oocytes to await fertilization, preventing the continuation of cell cycles and parthenogenesis after meiosis.

Once ovulated, eggs can either be fertilized and develop into embryo or they die within a short time. Fertilization causes the release of calcium from intracellular stores, which event is necessary and sufficient for egg activation. Consequently, calcium-dependent degradation of mitotic cyclins and Mos occurs, resulting in the inactivation of CSF and MPF, meiotic exit, and entry into the mitotic cell cycle. This allows development to proceed. Ovulated unfertilized eggs undergo a time-dependent quality loss, the process also known as postovulatory oocyte deterioration [7,8]. Accordingly, delayed egg fertilization results in progressive decrease of fertilization success in different frog, fish, and mammalian species [9-12]. Spontaneous activation of ovulated mammalian eggs has been implicated as a likely biochemical basis for the time-dependent decrease of the fertilization rate [13]. Also, the rapid loss of fertilization capacity of fish eggs during spawning has been linked to their spontaneous activation in aquatic environment [14].

The eggs from different species, such as starfish, mice and humans were shown to die by apoptosis within 24 hrs of ovulation if they are not fertilized [7,15,16]. The recent studies using starfish eggs implicate MAPK and calcium in triggering this process [17-19]. In this species, spontaneous egg activation and meiotic exit were shown to precede initiation of the apoptotic program. Still, in contrast to the well characterized apoptotic process in various somatic cells, apoptosis in eggs and oocytes is not well understood. Recently, cell-free extracts of *Xenopus *eggs that can support apoptosis [20,21] have been widely employed. These extracts are typically prepared from the eggs deposited by gonadotropin-stimulated animals. Paradoxically, since the time of their discovery in 1994, no cell-based apoptotic process recapitulated in these extracts has been identified. Although apoptosis in nutrient- or polyamine-deprived *Xenopus *oocytes and eggs has been well documented [22,23], no evidence has been presented that naturally laid frog eggs die by apoptosis. It has been suggested that the apoptosis in *Xenopus *egg extracts might reflect atretic oocyte degradation [24], however there is no experimental evidence for this suggestion. Notably, in mammalian species with internal fertilization, the oocytes that have matured but have not been ovulated are degraded apoptotically [25,26].

Here, we report that the majority of unfertilized *Xenopus *eggs are degraded by an apoptotic process within 48 hours after ovulation. In the absence of fertilization, under different environmental conditions, *Xenopus *eggs spontaneously exit meiotic arrest from 6 to 18 hours after ovulation. After the meiotic exit, eggs are degraded by an apoptotic process within following 24 hours. The hallmarks of this process include prominent morphological changes, cytochrome *c *release, caspase 3 activation, decrease of the intracellular ATP content, increase in ADP/ATP ratio, apoptotic nuclear morphology. At the late stages of egg degradation, prominent intracellular acidification and swelling can be observed. None of the apoptotic events could be detected in the eggs maintaining high activity of MPF and CSF and in the immature prophase-arrested oocytes, indicating that a maternal apoptotic program is evoked in frog oocytes upon maturation and executed after meiotic exit in unfertilized eggs. It takes several hours for apoptosis to develop in eggs after meiotic exit. In the same manner, apoptosis is initiated in metaphase-released *Xenopus *egg extracts only after their prolonged incubation at room temperature. Our findings suggest that the apoptosis in cell-free *Xenopus *egg extracts recapitulates the process of apoptotic degradation of unfertilized post-meiotic eggs.

## Results

### Unfertilized *Xenopus *eggs spontaneously exit meiotic arrest

**Figure 1:**
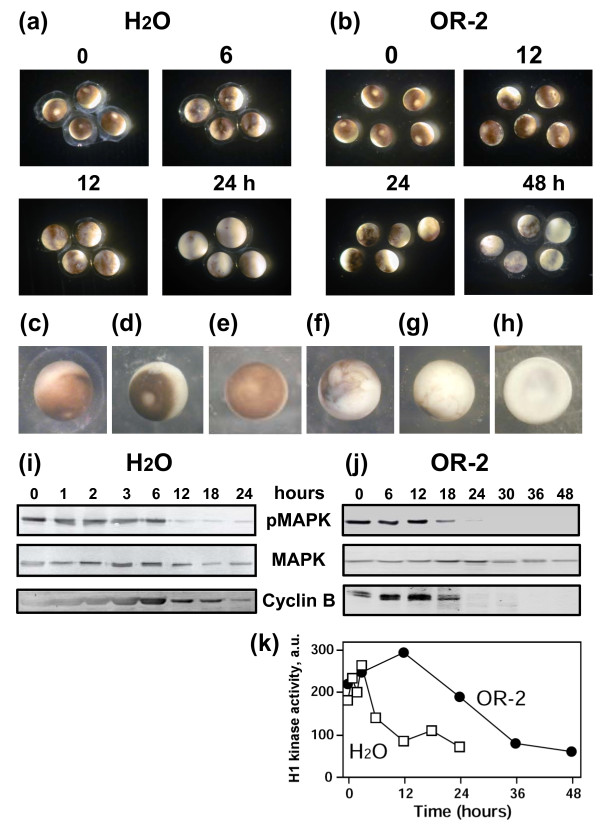
**Spontaneous meiotic exit in unfertilized *Xenopus *eggs**. Changes in the morphology of water-deposited **(a) **and OR-2 buffer-deposited **(b) **eggs, major morphological types of the unfertilized eggs **(c-h)**, MAPK dephosphorylation and cyclin B2 degradation **(i, j)**, and H1 kinase activity of Cdk1 in water-deposited and buffer-deposited eggs **(k) **are presented. Time after egg ovulation (hours) is indicated.

Immediately after their disposal, *Xenopus *eggs display quite uniform appearance. They are coated with jelly layer and have a prominent white spot in the animal hemisphere (Figure 1a, b, c). At that time, the eggs are arrested in metaphase, as it can be judged by the presence of cyclin B and phosphorylated MAPK (Figure 1i, j). Although some eggs experienced reversible cortical contraction (Figure 1d) characteristic of egg activation within the first several hours after ovulation, most of them remained arrested in metaphase for at least 6 hours in water and 12 hours in OR-2 buffer. Afterwards the unfertilized eggs spontaneously exited metaphase arrest, as witnessed by cyclin B degradation and MAPK dephosphorylation. Meiotic exit occurred earlier in water-deposited eggs than in buffer-deposited eggs (Figure 1i, j). By the time of meiotic exit, characteristic changes in the egg morphology could be detected. The white spot became hardly distinguishable and the eggs started to acquire marble-like appearance (Figure 1e, f). Subsequently, progressive decoloring of the pigment layer took place (Figure 1g). Presence of the jelly layer and different buffer composition have considerable effect on the timing of morphological changes and meiotic exit, notably, these events occurred faster in the eggs deposited in water (Table 1; Additional files 1, 2 and 3: Figures S1, S2, S3). Thus, under different environmental conditions unfertilized *Xenopus *eggs spontaneously exit meiotic arrest within 18 hours after ovulation. For the convenience of the following biochemical analysis, degradation of the unfertilized dejellied eggs deposited in OR-2 buffer has been investigated thereafter. Of note, a minor fraction of all eggs (typically, not exceeding 5% of the total population) experienced irreversible cortical contraction leading to the fast egg whitening and swelling within just 2 hours (Figure 1h). These eggs were excluded from the detailed biochemical analysis (see Discussion).

**Table 1:** Timing of morphological and biochemical changes in the unfertilized *Xenopus *eggs deposited into different buffers.

Egg environment	Change in egg morphology	Meiotic exit	Caspase acti- vation	Increase in egg diameter
**Water (j)**	within 6 h	within 12 h	18-36 h	12-18 h
**DB buffer (d)**	within 12 h	within 18 h	24-36 h	24-36 h
**OR-2 (j)**	within 12 h	within 18 h	30-48 h	24-36 h
**OR-2 (d)**	within 12 h	within 18 h	24-36 h	24-36 h

(d) and (j) denote dejellied and jelly-coated eggs, respectively.

### Apoptotic events in unfertilized *Xenopus *eggs

**Figure 2:**
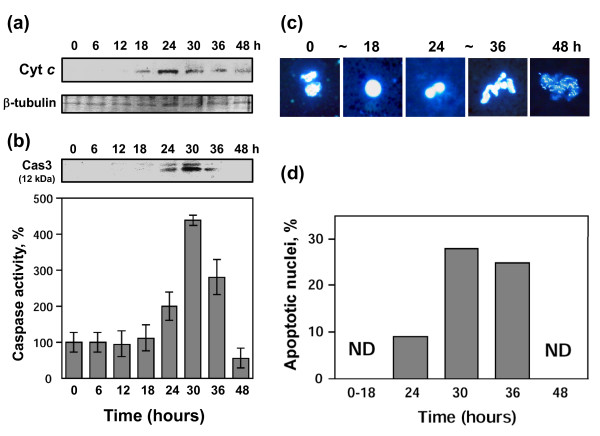
**Features of classical apoptosis in unfertilized *Xenopus *eggs**. Freshly squeezed eggs obtained after hCG injection were dejellied and placed into OR-2 buffer over the indicated times. Cytochrome *c *release **(a)**, caspase 3 activation **(b)**, apoptotic nuclear morphology **(c)**, and quantification of morphology scores **(d) **are shown. Immunoblotting with anti-β tubulin (lower panel in **(a)**) represents the loading control. The upper panel in **(b) **shows blotting of egg extracts with anti-caspase 3 antibody, whereas the lower panel presents data of the fluorescent caspase 3 assay, carried out as described in "Methods". Bars in panel **(b) **indicate SD of five to eight measurements using eggs obtained from three different female frogs. About one hundred nuclei were observed in **(c) **and **(d)**.

**Figure 3:**
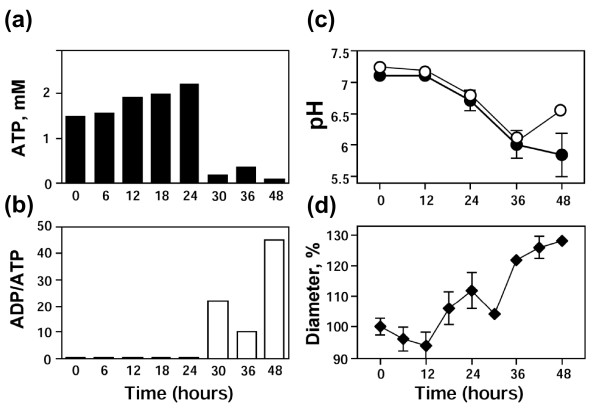
**Cell death events in unfertilized *Xenopus *eggs**. Intracellular ATP content **(a)**, ADP/ATP ratio **(b)**, intracellular pH **(c)**, and egg diameter **(d) **were monitored over 48 hours after ovulation. Intracellular pH in panel **(c) **was measured using spectrometric and fluorescent assays (closed and open circles, respectively). Bars in panel **(c) **represent the range of pH readings taken by two persons in double-blind trials and data in panel **(d) **are means ± SD obtained by measurement of five eggs.

Besides the beginning morphological changes, no other features of egg degradation could be detected at the time of meiotic exit. No significant changes in the level of cytochrome *c *released from mitochondria, caspase activity, intracellular ATP content, ADP/ATP ratio, and intracellular pH have been observed during the first 12 hours after ovulation (Figures 2, 3). The stability of the egg size evidences that the mechanisms of cellular osmotic homeostasis operate properly over that time (Figure 3d). However, the dramatic biochemical changes, indicative of unfolding apoptotic process, developed in the eggs after meiotic exit. By 24-36 hours after ovulation, the eggs displayed significantly elevated caspase 3 activity and they abundantly released cytochrome *c *from mitochondria (Figure 2a, b; Table 2). The cell-free extracts prepared from these eggs induced apoptotic nuclear morphology in the added demembranated sperm nuclei (Figure 2c, d). These features are characteristics of the classical apoptotic process. Furthermore, depletion of intracellular ATP, increase in ADP/ATP ratio, and significant intracellular acidification could be observed in the eggs by 30-36 hours after ovulation (Figure 3a, b, c). The prominent increase in the intracellular egg volume by that time (Figure 3d) is indicative of the irreversible loss of plasma membrane integrity that essentially defines the terminal stage of cell death.

**Table 2:** Timing of apoptotic events in the ovulated *Xenopus *eggs and *in vitro*-matured oocytes.

Events in eggs	Control eggs	PG-treated oocytes	Ionophore- activated eggs	Roscovitine- treated eggs
**Meiotic exit**	within 18 h	within 24 h	within 45 min	within 6 h
**Cytochrome c release**	24 h	24-36 h	18 h	ND*
**Caspase 3/7 activation**	24-36 h	48-60 h	18-24 h	18-36 h
**ATP decrease**	30 h	60 h	24 h	24 h
**ADP/ATP increase**	30 h	60 h	24 h	24 h
**Drop in intracellular pH (< 6.0)**	36 h	60 h	36 h	ND*
**Increase in egg diameter**	36 h	60 h	36 h	24 h

*not determined

Of note, prominent intracellular acidification could be consistently detected in the eggs by 36 hours using both spectrometric and fluorescent assays (Figure 3c, closed and open circles, respectively). However, overestimation of the pH value in late apoptotic egg lysates (48 hours) was evident with the fluorescent assay due to, presumably, elevated pH-independent fluorescence of BCECF in these lysates. The spectrometric pH assay was devoid of this drawback, so, it was mainly employed in the following experiments.

### Single-cell analysis of unfertilized *Xenopus *eggs

**Figure 4:**
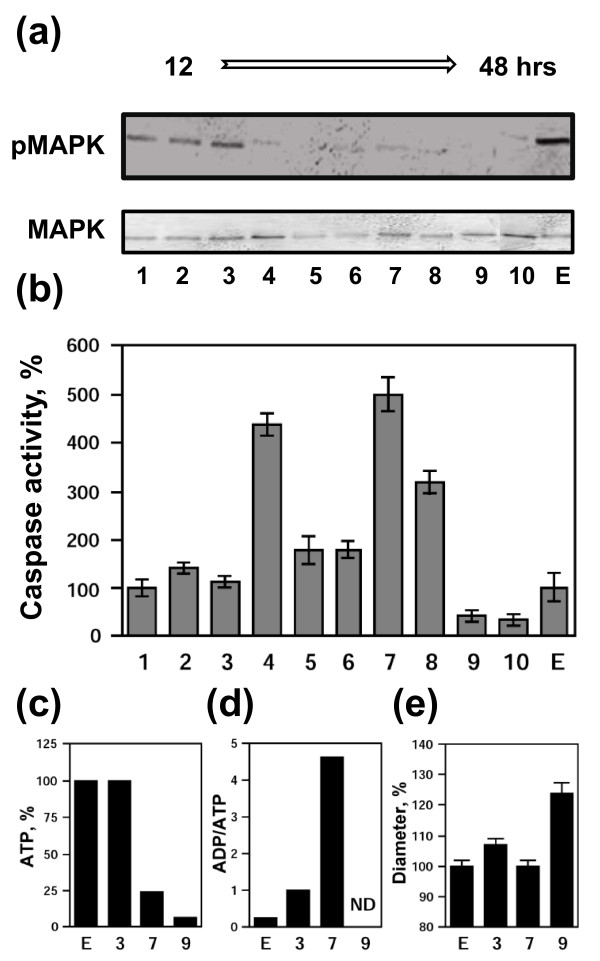
**Single-cell analysis of unfertilized *Xenopus *eggs**. MAPK phosphorylation state **(a) **and caspase 3 activity **(b) **were determined in the single unfertilized eggs #1-10 taken at different times (12 - 48 hours) after their deposition into OR-2 buffer. Eggs #1-3 were collected within 12 hours of ovulation, eggs #4-8 - within 18-36 hours, and eggs #9-10 - at 48 hours after ovulation. The control egg E was withdrawn immediately after its deposition. Relative intracellular ATP content **(c)**, ADP/ATP ratio **(d)**, and egg diameter **(e) **were determined for the eggs #3, 4, 9, and E. Bars in **(b) **and **(e) **represent the range of duplicate measurements. The total number of eggs taken for the single-cell analysis was 39, among them, 10 eggs were collected within 12 hours of ovulation, 22 eggs - within 18-36 hours, and 7 eggs - at 48 hours after ovulation.

To better understand the sequence of events in a potentially heterogeneous population of unfertilized *Xenopus *eggs, biochemical analysis of the individual eggs has been carried out. The eggs collected at 12 hours after ovulation (Figure 4; eggs #1-3) were found to be arrested in the metaphase, and no metaphase-arrested eggs were present in egg population afterwards, as judged by the phosphorylation status of MAPK (Figure 4a). The level of caspase 3 activity in these eggs was close to that in the control egg collected immediately after ovulation (Figure 4b, egg E). Also, the content of intracellular ATP, ADP/ATP ratio, and egg size were similar to the control (Figure 4c, d, e). Cell-to-cell variation of the caspase activity at that time was rather low, reflecting high homogeneity of the egg population. However, the eggs collected at 18-36 hours after ovulation (eggs #4-8) displayed elevated caspase activity, and the degree of caspase activation in the individual eggs varied significantly in the range between 160% and 500% (Figure 4b). These figures agree well with about four-fold magnitude of caspase activation observed in the bulk-scale experiments, which measure average values for egg population (Figure 2b). The significant loss of intracellular ATP and increase in ADP/ATP ratio were evident at 36 hours after ovulation (egg #7, Figure 4c, d). However, egg diameter did not yet alter significantly at that time (Figure 4e). Finally, the eggs collected at 48 hours after ovulation (eggs #9, 10) displayed very low caspase activity and they were virtually depleted of ATP. Their diameter was drastically increased, indicating the loss of cellular osmotic homeostasis. These findings are largely consistent with the data obtained in the bulk-scale experiments. In addition, the single-cell analysis revealed a significant egg-to-egg variation of the caspase activation, suggesting considerable heterogeneity of the apoptotic response in the analyzed egg population.

### Early meiotic exit promotes accelerated apoptosis in *Xenopus *eggs

**Figure 5:**
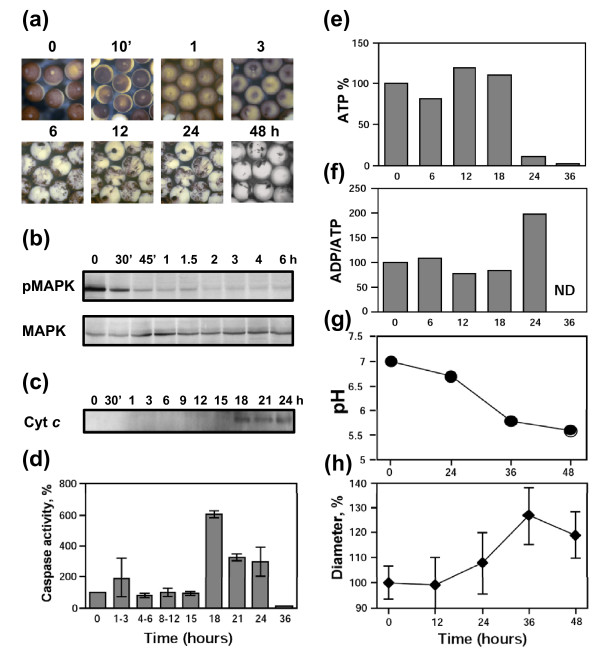
**Apoptotic degradation of ionophore-treated *Xenopus *eggs**. Freshly squeezed dejellied eggs were placed into OR-2 buffer, treated with 1 μM calcium ionophore A23187 for 5 min and monitored over indicated times. Egg morphology **(a)**, MAPK activation state **(b)**, cytochrome *c *release **(c)**, caspase 3 activity **(d)**, intracellular ATP content **(e)**, ADP/ATP ratio **(f)**, intracellular pH **(g)**, and egg diameter **(h) **have been monitored. In panel **(d)**, the data of three to six measurements in two independent experiments are shown. Bars in panel **(h) **represent SD of the mean obtained by measurement of three to five eggs.

Meiotic exit was found to precede apoptosis in the unfertilized *Xenopus *eggs (Figures 1, 2). Moreover, apoptotic events could not be detected in the eggs arrested at the meiotic metaphase (Figure 4). These data suggested that meiotic arrest may act to prevent apoptosis in the unfertilized eggs. If this suggestion is right, the early meiotic exit should promote accelerated egg apoptosis and degradation. To test this lead, freshly-deposited *Xenopus *eggs were artificially activated with the calcium ionophore A23187. This treatment induced robust egg activation and meiotic exit within 45 minutes (Figure 5a, b; Table 2). However, no signs of unfolding apoptotic process have been observed in the eggs over the next 15 hours following activation. Cytochrome *c *release and caspase activation in the eggs could be detected only in 18 hours after the ionophore administration. Notably, these events occurred earlier and they were more robust in the ionophore-treated eggs than in the naturally laid eggs (Figure 5c, d; Table 2). Also, the earlier ATP decrease and ADP/ATP ratio increase were evident in the ionophore-activated eggs (Figure 5e, f; Table 2).

Presently, it is unknown if calcium rise is involved in releasing the naturally laid *Xenopus *eggs from meiotic arrest, so, the results with calcium ionophore cannot be immediately interpreted as physiologically relevant. To directly support a role of the cell cycle machinery in preventing apoptosis, we examined whether the specific Cdk inhibitor roscovitine can accelerate apoptosis in aging *Xenopus *eggs. We have found that similarly to calcium ionophore, roscovitine promoted earlier meiotic exit in these cells; cyclin and Mos degradation and MAPK dephosphorylation occurred within 6 hours of drug administration (Additional file 4: Figure S4). Importantly, the earlier meiotic exit was accompanied by accelerated apoptosis; faster morphological changes, caspase activation, ATP depletion, ADP/ATP ratio increase, and egg swelling have been observed in the roscovitine-treated eggs (Table 2; Additional file 4: Figure S4).

Altogether, the results obtained demonstrate that earlier meiotic exit is associated with accelerated and more robust apoptotic degradation of unfertilized *Xenopus *eggs. The data provide a basis to infer cause-and-effect relationships between meiotic exit and apoptosis, suggesting an anti-apoptotic role for meiotic metaphase arrest. However, the fact that apoptosis does not unfold in the eggs immediately after meiotic exit indicates that some other factors also contribute to inhibition of the apoptotic program in these cells (see Discussion).

### *In vitro *maturation of *Xenopus *oocytes initiates maternal apoptotic program

**Figure 6:**
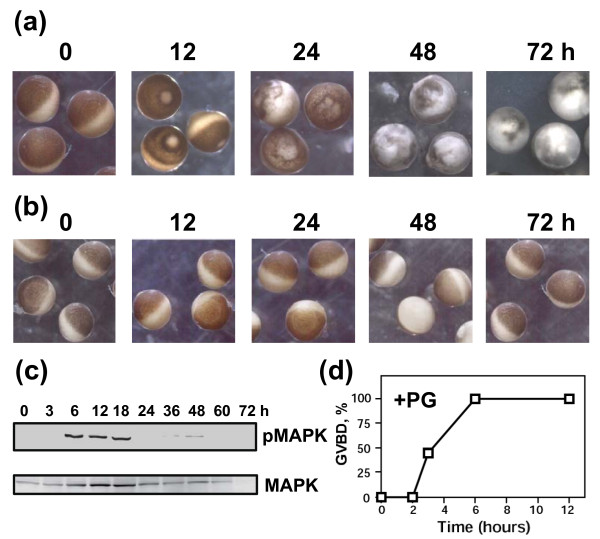
**Stability of progesterone-treated and intact *Xenopus *oocytes**. Defollliculated OR-2 buffer-deposited oocytes were treated with 10 μM progesterone or left untreated and monitored over 72 hours. Morphology of the progesterone-treated **(a) **and -untreated **(b) **oocytes, MAPK phosphorylation state **(c)**, and time course of GVBD **(d) **after progesterone addition.

**Figure 7:**
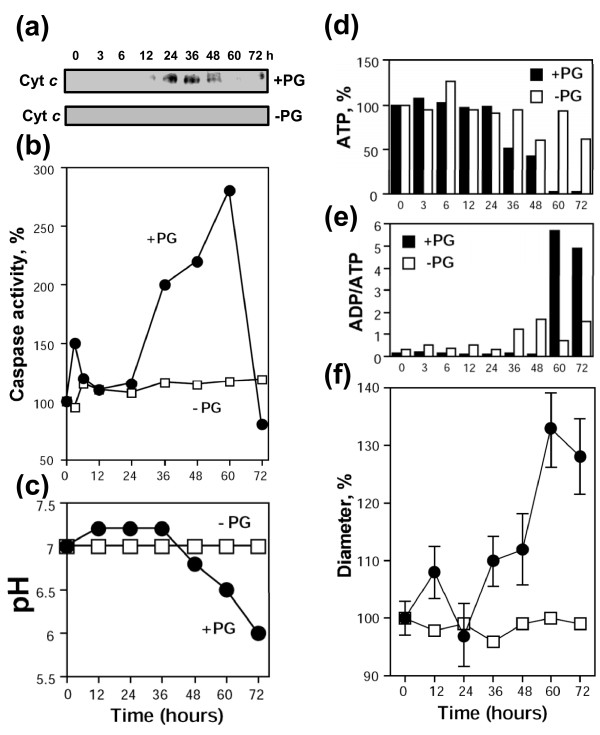
**Apoptotic degradation of progesterone-treated *Xenopus *oocytes**. Cytochrome *c *release **(a)**, caspase 3 activation **(b)**, intracellular pH **(c)**, intracellular ATP content **(d)**, ADP/ATP ratio **(e)**, and egg diameter **(f)**. Bars in panel **(f) **indicate SD of the mean obtained by measurement of five eggs.

The data presented above indicate that the majority of unfertilized *Xenopus *eggs die by an apoptotic process within 48 hours of ovulation. On the other hand, immature fully grown *Xenopus *oocytes of stage VI can rest in frog ovaries over several months. These facts imply that the apoptotic program may be initiated in the oocytes upon meiotic maturation. To confirm this suggestion, we compared out-of-body stability of the surgically-removed defolliculated *Xenopus *oocytes and eggs obtained from these oocytes by *in vitro *maturation. The results of these experiments demonstrated that the oocytes remained stable outside of the animal's body in the OR-2 media for at least 72 hours (Figure 6b). None of the cell death events, such as cytochrome *c *release, caspase activation, intracellular acidification, and swelling could be observed in the resting oocytes over that period (Figure 7a, b, c, f). However, some moderate decrease of the intracellular ATP content and increase in ADP/ATP ratio, related, probably, to oocyte ageing, was evident (Figure 7d, e). On the other hand, the studied apoptotic events manifested profoundly in the oocytes subjected to progesterone treatment. The hormone promoted meiotic oocyte maturation and cell cycle transition from prophase to metaphase, as judged by MAPK activation and GVBD (Figure 6c, d). Afterwards, similarly to the naturally laid eggs, the *in vitro *matured oocytes experienced spontaneous meiotic exit (Figure 6c) accompanied by the dramatic changes in the egg morphology (Figure 6a). All the cell death events observed in the ovulated eggs, such as cytochrome *c *release, caspase activation, intracellular acidification, loss of intracellular ATP, increase in ADP/ATP ratio, and swelling, could also be detected in the *in vitro *matured oocytes, albeit with some delay (Figure 7; Table 2). Altogether, the data of the *in vitro *maturation experiments demonstrate that progesterone renders *Xenopus *oocytes susceptible to apoptosis. In accordance with the results obtained with ovulated eggs (Figures 1, 2, 3, 4, 5), apoptosis unfolds in the *in vitro *matured oocytes after meiotic exit.

## Discussion

The main finding of this study is that unfertilized frog eggs die by apoptosis following meiotic exit. A plethora of classical apoptotic events, such as cytochrome *c *release, caspase activation, apoptotic nuclear morphology, decrease of intracellular ATP, increase in ADP/ATP ratio, and intracellular acidification have been observed in the unfertilized eggs of the African clawed frog *Xenopus laevis*. Notably, previous studies failed to reveal apoptotic features in the naturally laid intact *Xenopus *eggs. For instance, cytochrome *c *release could not be detected in the intact metaphase II arrested eggs over 18 hours of ovulation [22]. More recently, real-time monitoring of caspase activity in *Xenopus *oocytes and early embryos using near-infrared fluorescence failed to detect caspase activation in the progesterone-matured oocytes over 24 hours and concluded that death in the mature oocytes arrested in meiosis II proceeds by a process different from classical apoptosis [27]. The reason for the failed detection of the caspase activation in the unfertilized frog eggs seems to be the greatly delayed onset of apoptosis in these cells. Even when the eggs appeared, by morphological criteria, to be dying, there was no indication of caspase activation in these eggs (Figures 1, 2). Accordingly, other events of classical apoptosis, such as cytochrome *c *release, apoptotic nuclear morphology, loss of intracellular ATP, increase in ADP/ATP ratio, and intracellular acidification could not be detected in the unfertilized *Xenopus *eggs until 24 hours after ovulation (Figures 2, 3). Eventually, after the delay, the eggs degraded by a well-defined apoptotic process within 48 hours after ovulation (Figures 2, 3, 4). In accordance with these results, the most recent study published after submission of the present paper reported that ovulated unfertilized *Xenopus *eggs recovered in the external medium died by apoptosis within 72 hours [28].

Our data suggest that meiotic arrest in frog eggs is one of the factors responsible for the delay in the execution of apoptotic cell death. Under different environmental conditions, unfertilized *Xenopus *eggs were found to spontaneously exit meiotic arrest (Table 1; Additional files 1, 2, 3: Figures S1, S2, S3). Meiotic exit precedes apoptosis and none of the investigated apoptotic events could be detected in metaphase-arrested eggs (Figures 2, 4). In addition, the early meiotic exit induced by calcium ionophore or roscovitine was found to significantly accelerate apoptosis onset (Figure 5, Additional file 4: Figure S4; Table 2). Markedly, synchronization of meiotic exit by the ionophore and roscovitine induced more robust and synchronous apoptotic process, suggesting causality between meiotic arrest and apoptosis. In connection with these data, the earlier meiotic exit in water, as compared to that in DB buffer or OR-2 media, was also accompanied by the accelerated egg apoptosis (Figure 1; Table 1). Thus, meiotic exit seems to be a prerequisite for execution of the apoptotic program, since (i) it precedes apoptosis, (ii) none of the apoptotic events can be observed in the eggs maintaining high activity of MPF and CSF, and (iii) apoptosis in the unfertilized frog eggs is accelerated upon early meiotic exit.

Previously, it has been demonstrated that mature eggs are much more sensitive to apoptotic insult than prophase oocytes [22]. Also, the experiments involving *in vitro *reconstitution of apoptosis in cell-free extracts of *Xenopus *eggs suggested that the extracts arrested in interphase, but not in metaphase were susceptible to an apoptotic program leading to caspase activation [29,30]. Although apoptotic extracts are usually prepared from the eggs arrested in meiotic metaphase II, egg lysis by centrifugation in the process of extract preparation causes calcium release, which promotes meiotic exit and progression into interphase. The meiotic extracts prepared in the presence of calcium chelators are markedly refractory to apoptosis [29]. It was shown that the MAPK pathway active in the metaphase-arrested egg extracts renders them resistant to apoptosis [30]. Caspase 9 phosphorylation at Thr125 targeted by ERK MAPK *in vitro *was shown to be sufficient to block caspase 9 processing and subsequent caspase 3 activation [31]. It has also been reported that phosphorylation of caspase 9 by Cdk1/cyclin B1 protects mitotic cells against apoptosis [32]. In addition, Cdk1/cyclin B1 was shown to suppress apoptosis during mitosis through interdomain phosphorylation of caspase 2 within an evolutionarily conserved sequence at Ser 340 [33]. The additional experiments using specific inhibitors and activators of MAPK and Cdk1 are required to reveal differential anti-apoptotic engagement of CSF and MPF in the unfertilized *Xenopus *eggs.

Suppressing apoptotic program in mature frog eggs should serve the purpose of producing the largest possible number of gametes available for fertilization. This situation is quite reminiscent of the apoptotic induction in star fish eggs, where meiotic arrest effectively blocks apoptosis [16,18]. It was shown that induction of apoptosis in starfish eggs requires spontaneous MAPK inactivation followed by p38MAPK activation [18,19]. Moreover, before the inactivation, MAPK should be activated for a definite period, during which eggs develop competence to die. Similarly, mature *Xenopus *oocytes should also be arrested at metaphase II with high activity of MAPK and Cdk1 before they can initiate apoptosis. Immature *Xenopus *oocytes, which have low activity of these meiotic regulators, were found to be markedly refractory to apoptosis (Figure 6). Previously, microinjection of cytochrome *c *into immature *Xenopus *oocytes was shown to reliably induce apoptosis [34], suggesting pre-cytochrome *c *protection from apoptosis in these cells. In addition, the finding that mature eggs are much more sensitive to apoptotic insult caused by cytochrome *c *microinjection than prophase oocytes [22] also indicates the existence of post-cytochrome c protection from apoptosis in the oocytes. In the future studies, it would be interesting to identify, at which stage of meiotic maturation frog oocytes develop competence to apoptosis.

Remarkably, although apoptotic protection conferred by meiotic arrest is lost at egg activation, unfertilized post-meiotic eggs are not engaged into apoptosis immediately after meiotic exit. It takes several hours to release cytochrome *c *and fully activate caspases after either spontaneous or calcium ionophore-initiated meiotic exit (Figures 1, 2, 7). Similarly, interphase *Xenopus *egg extracts do not release cytochrome *c *until they have been incubated at room temperature for prolonged periods [20], suggesting that some mechanisms must act to prevent apoptosis during that time. Presumably, the same mechanisms work to suppress apoptosis after egg fertilization and during early embryonic divisions. It has been suggested that apoptosis is prevented during the early cleavages in the *Xenopus *embryo by maternally encoded apoptotic inhibitors [35,36], however their identity has not been disclosed.

Notably, the apoptotic scenario revealed in this study is not the only way of egg degradation. Some minor fraction of ovulated eggs (typically, not exceeding 5% of total population) experience robust and irreversible cortical contraction. Although this phenomenon occurs most often within the first 3 hours of ovulation, it can also be observed at later times. Irreversible cortical contraction is associated with excessive egg activation (so called, hyperactivation), leading to the fast egg whitening and swelling within just one hour (Figure 1h). Egg hyperactivation is considered to be a pathological process [37]. Evidently, it is quite different from the normal egg activation, so the hyperactivated eggs were excluded from the present biochemical analysis. It should be noted that although normally the percent of hyperactivated cells in a batch of ovulated eggs is quite low, it varies greatly according to season and individual animals. Some batches of poor quality, especially those deposited in the summer time, may be composed almost entirely of the hyperactivated eggs.

## Conclusions

The present study reveals that: (i) unfertilized *Xenopus *eggs die by apoptosis if they are not fertilized, (ii) apoptosis unfolds in the eggs following meiotic exit, (iii) a maternal apoptotic program is evoked in frog oocytes upon meiotic maturation, and (vi) meiotic exit is a prerequisite for execution of the apoptotic program in *Xenopus *eggs. These findings make the unfertilized frog eggs a very attractive and feasible model for apoptotic studies. In addition, our results suggest that the well-established apoptosis in cell-free *Xenopus *egg extracts recapitulates the process of apoptotic degradation of unfertilized post-meiotic eggs.

## Methods

### Materials

Female frogs of *Xenopus laevis *were purchased from Hamamatsu Seibutsu Kyozai (Hamamatsu, Japan) and maintained in dichloride tap water at ambient temperature (21-23°C). Microcon centrifugal filter devices (MWCO 100 kDa) were from Millipore (Billerica, MA). pH test paper for the interval 5.4 - 7.0 was from Advantec Toyo Roshi (Japan). Fluorescent pH indicator BCECF and Cdk inhibitor roscovitine were purchased from Wako (Tokyo, Japan). Polyclonal anti-MAPK and anti-pMAPK antibodies were from Cell Signaling (Beverly, MA). Anti-caspase 3 Ab-4 antibody was from Thermo Fisher Scientific (Waltham, MA). Rabbit polyclonal anti-cyclin B2 antibody, anti-β tubulin antibody, anti-cytochrome *c *antibody, and alkaline phosphatase-conjugated goat polyclonal antibody against rabbit IgG were from Santa Cruz (Santa Cruz, CA). Progesterone, histone H1, and PKA inhibitor peptide were obtained from Sigma (St. Louis, MO) and human chorionic gonadotropin (hCG) was from Teikoku Zoki (Tokyo, Japan). Fluorogenic caspase-3 substrate IV was from Calbiochem (La Jolla, CA). Bioluminescent ApoSENSOR ADP/ATP ratio assay kit was purchased from BioVision (Mountain View, CA). Other chemicals were from Nacalai Tesque (Kyoto, Japan), Wako (Osaka, Japan), or Sigma.

### Animal treatment, oocyte and egg isolation

This study was conducted according to the Kobe University Animal Experimentation Regulation. All the experiments with oocytes and eggs were carried out at the ambient temperature of 21-23°C. To obtain eggs, frogs were injected hypodermically with 500 IU per animal of hCG in the dorsal lymph sac. Ovulation began in 8 to 10 hours after the hCG injection. Eggs were gently squeezed into DeBoer's buffer (DB) containing 110 mM NaCl, 1.3 mM KCl and 0.44 mM CaCl_2_, adjusted to pH 7.2 by addition of NaHCO_3_. The jelly layer was removed from eggs by incubation with the twofold volume of 2% cysteine in DB, pH 8.0, for 3-6 minutes. After the treatment, eggs were washed extensively with DB solution.

To obtain oocytes, frogs were anesthetized on ice followed by rapid decapitation, then ovaries were surgically removed and placed into OR-2 solution containing 82.5 mM NaCl, 2.5 mM KCl, 1 mM CaCl_2_, 1 mM MgCl_2_, 1 mM Na_2_HPO_4_, 5 mM HEPES, pH 7.6. Ovaries were manually dissected into clumps of 50-100 oocytes and extensively washed with OR-2 solution. Clumps of oocytes were treated with 0.5 mg/ml collagenase (280 U/mg) in OR-2 at 23°C for 3-4 hours by shaking at 60 rpm. Oocytes were extensively washed in OR-2 solution and left for stabilization over 4 h. Undamaged defolliculated oocytes of stage VI were manually selected and used in the experiments.

*In vitro *oocyte maturation was induced by addition of 10 μM progesterone and monitored by appearance of a white spot on the animal hemisphere of oocytes. Eggs were artificially activated by 5-min treatment with 1 μM A23187. After the activation treatment, eggs were washed twice with DB solution.

### Detection of nuclear morphology

Detection of apoptotic nuclear fragmentation was carried out by monitoring the morphology of demembranated sperm nuclei in cell-free egg extracts prepared essentially as described previously [38]. Briefly, dejellied eggs were washed extensively with the extract buffer (XB), containing 100 mM KCl, 0.1 mM CaCl_2_, 1 mM MgCl_2_, 50 mM sucrose, and 10 mM potassium HEPES, pH 7.7, then transferred into centrifuge tubes containing XB buffer plus 100 μg/ml cytochalasin B (Sigma) and 10 μg/ml each leupeptin, pepstatin, and chymostatin. Eggs were packed by low-speed centrifugation at 400 g. All buffer was removed from the top of the tubes and the eggs were crushed by centrifugation at 10, 000 g for 15 min at 4°C. The cytoplasmic layer was collected and supplemented with 1/20 volume of energy mix (150 mM creatine phosphate, 20 mM ATP, 20 mM MgCl_2_, 2 mM EGTA, pH 7.7). Demembranated sperm nuclei were prepared as described earlier [39] and added to the extracts to a final concentration of 10^5 ^nuclei per ml. After 3-hour long incubation at RT, nuclear morphology was observed by fluorescent microscopy, as described previously [38].

### Immunoblotting

Protein samples were separated by SDS PAGE using 10% or 15% polyacrylamide gels and transferred to polyvinulidene difluoride membranes using a semidry blotting device (BioRad). Membranes were blocked with T-TBS buffer (20 mM Tris-HCl, pH 7.5, 150 mM NaCl, and 0.05% Tween 20) containing 3 mg/ml bovine serum albumin and incubated at RT for 2 hours with 500-fold diluted anti-caspase 3 antibody, or 200-fold diluted anti-MAPK or anti-β tubulin antibodies, or 100-fold diluted anti-phospho MAPK, anti-cyclin B2, or anti-cytochrome *c *antibodies. After washing, the membranes were treated with 1000-fold diluted alkaline phosphatase-conjugated goat polyclonal antibody against rabbit IgG. The immune complexes were detected by color development catalyzed by alkaline phosphatase conjugated to the secondary antibody.

### Protein kinase assay

Cdk1 activity in the eggs was assessed by specific phosphate incorporation into histone H1 protein substrate. The reaction mixture of protein kinase assay (20 μl) contained 50 mM Tris-HCl, pH 7.5, 10 mM MgCl_2_, 1 mg/ml histone H1, 2 μM protein kinase A inhibitor peptide, 100 μM [γ-^32^P] ATP and 5 μl of egg lysate (equivalent to 0.4 egg). Samples were incubated at 25°C for 15 min, then the reaction was stopped by the addition of concentrated Laemmli's buffer [40]. The reaction products were separated by SDS-PAGE, and radioactive bands of H1 were visualized by an image analyzer (BAS2500, FUJI Film).

### Caspase 3 activity assay

Eggs were disrupted by pipetting in 20-fold excess of the ice-cold OR-2 buffer followed by centrifugation to remove insoluble material. To measure caspase 3 activity, 1-μl samples of lysates were incubated with 10 μM fluorogenic substrate peptide, caspase substrate IV, in the assay buffer for 3 hours at room temperature with a soft shaking at 100 rpm. Fluorescence was then measured using the LAS1000 plus Luminescent Image Analysis System (FUJIFILM, Tokyo, Japan).

### Cytochrome *c *release assay

Eggs were disrupted by pipetting in 20-fold excess of the ice-cold cytochrome *c *assay buffer (250 mM sucrose, 1 mM EDTA, 30 mM Tris, pH 7.5), which supports mitochondrial integrity. The lysates were filtered through the membrane filters (MWCO 100 kDa). The filtrates were subjected to SDS PAGE on 15% gels and analyzed by immunoblotting with anti-cytocrome *c *antibody, as described above.

### Intracellular pH measurements

Fifty to hundred dejellied eggs were washed extensively with NANOpure water to remove buffer. Eggs were packed by low-speed centrifugation at 400 g and then lysed by centrifugation at 10, 000 g, as described above. Ten to twenty microliters of cytoplasmic layer were spotted onto the Advantec pH indicator paper (measurement range 5.4-7.0) and read by two persons in double-blind trials.

Alternatively, the fluorescent pH indicator BCECF at 1 μM concentration was used to measure pH in the egg lysates prepared by the above method. The pH calibration curve was built over the pH range of 6.0 - 8.0 in the presence of egg lysate. Sample fluorescence was quantified using the LAS1000 plus Luminescent Image Analysis System.

### Other methods

Protein content in the samples was determined spectrophotometrically using a protein assay kit (Bio-Rad). Measurement of intracellular ATP and ADP/ATP ratio was carried out using the ApoSENSOR assay kit from from BioVision according to the manufacturer's manual. Egg diameter was measured by determining the geometric mean of two perpendicular measurements taken in the microscopic egg image.

## Abbreviations

Cdk: cyclin-dependent kinase; CSF: cytostatic factor; GVBD: germinal vesicle breakdown; MAPK: mitogen-activated protein kinase; MPF: maturation promoting factor.

## Authors' contributions

AAT designed the research, performed the experiments and wrote the paper. SI performed the experiments and analyzed data. TI and YF participated in the design of the study and contributed critical comments and suggestions. All authors read and approved the final manuscript.

## Supplementary Material

Additional file 1 **Figure S1**. Degradation of unfertilized jelly-coated *Xenopus *eggs deposited into water. **(a) **Caspase activation and **(b) **egg diameter. Data in panel **(a) **are means ± SD of three measurements, data in panel **(b) **were obtained by measuring five eggs. Click here for file

Additional file 2 **Figure S2**. Degradation of unfertilized jelly-coated *Xenopus *eggs deposited into OR-2 media. **(a) **Caspase activation and **(b) **egg diameter. Data in panel **(a) **are means ± SD of three measurements, data in panel **(b) **were obtained by measuring three to five eggs. Click here for file

Additional file 3 **Figure S3**. Degradation of unfertilized dejellied *Xenopus *eggs deposited into DB buffer. **(a) **Changes in egg morphology, **(b) **MAPK dephosphorylation, **(c) **caspase activation, and **(d) **egg diameter. Data in panel **(d) **were obtained by measuring five eggs. Click here for file

Additional file 4 **Figure S4**. Apoptotic degradation of roscovitine-treated *Xenopus *eggs. Freshly squeezed dejellied eggs were placed into OR-2 buffer and treated with 50 μM roscovitine. Egg morphology **(a)**, Mos, cyclin B2 levels and MAPK activation state **(b)**, caspase 3 activity **(c)**, intracellular ATP content **(d)**, ADP/ATP ratio **(e)**, and egg diameter **(f) **have been monitored at the indicated times. Bars in panel **(f) **represent SD of the mean obtained by measurement of seven eggs. Click here for file
