# Supplementary material for: Unfertilized frog eggs die by apoptosis following meiotic exit
Source: BMC Cell Biol. 2011 Dec 23;12:56. doi: 10.1186/1471-2121-12-56 (PMC3268744; doi:10.1186/1471-2121-12-56)
Supplement: Additional file 2 — Figure S2. Degradation of unfertilized jelly-coated Xenopus eggs deposited into OR-2 media. (a) Caspase activation and (b) egg diameter. Data in panel (a) are means ± SD of three measurements, data in panel (b) were obtained by measuring three to five eggs. [file 1471-2121-12-56-S2.PDF]

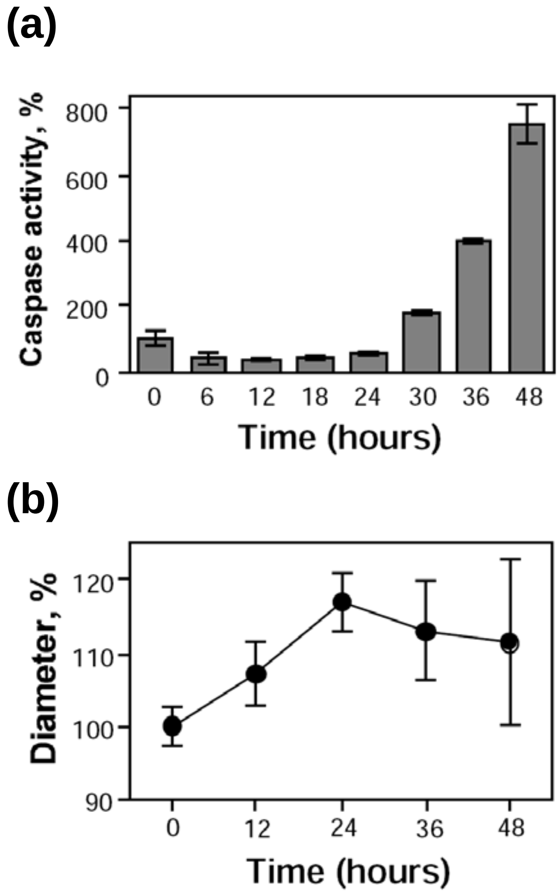

Figure S2. Degradation of unfertilized jelly-coated *Xenopus* eggs deposited into OR-2 media. **(a)** Caspase activation and **(b)** egg diameter. Data in panel **(a)** are means ±SD of three measurements, data in panel **(b)** were obtained by measuring three to five eggs.
