# Supplementary material for: Unfertilized frog eggs die by apoptosis following meiotic exit
Source: BMC Cell Biol. 2011 Dec 23;12:56. doi: 10.1186/1471-2121-12-56 (PMC3268744; doi:10.1186/1471-2121-12-56)
Supplement: Additional file 3 — Figure S3. Degradation of unfertilized dejellied Xenopus eggs deposited into DB buffer. (a) Changes in egg morphology, (b) MAPK dephosphorylation, (c) caspase activation, and (d) egg diameter. Data in panel (d) were obtained by measuring five eggs. [file 1471-2121-12-56-S3.PDF]

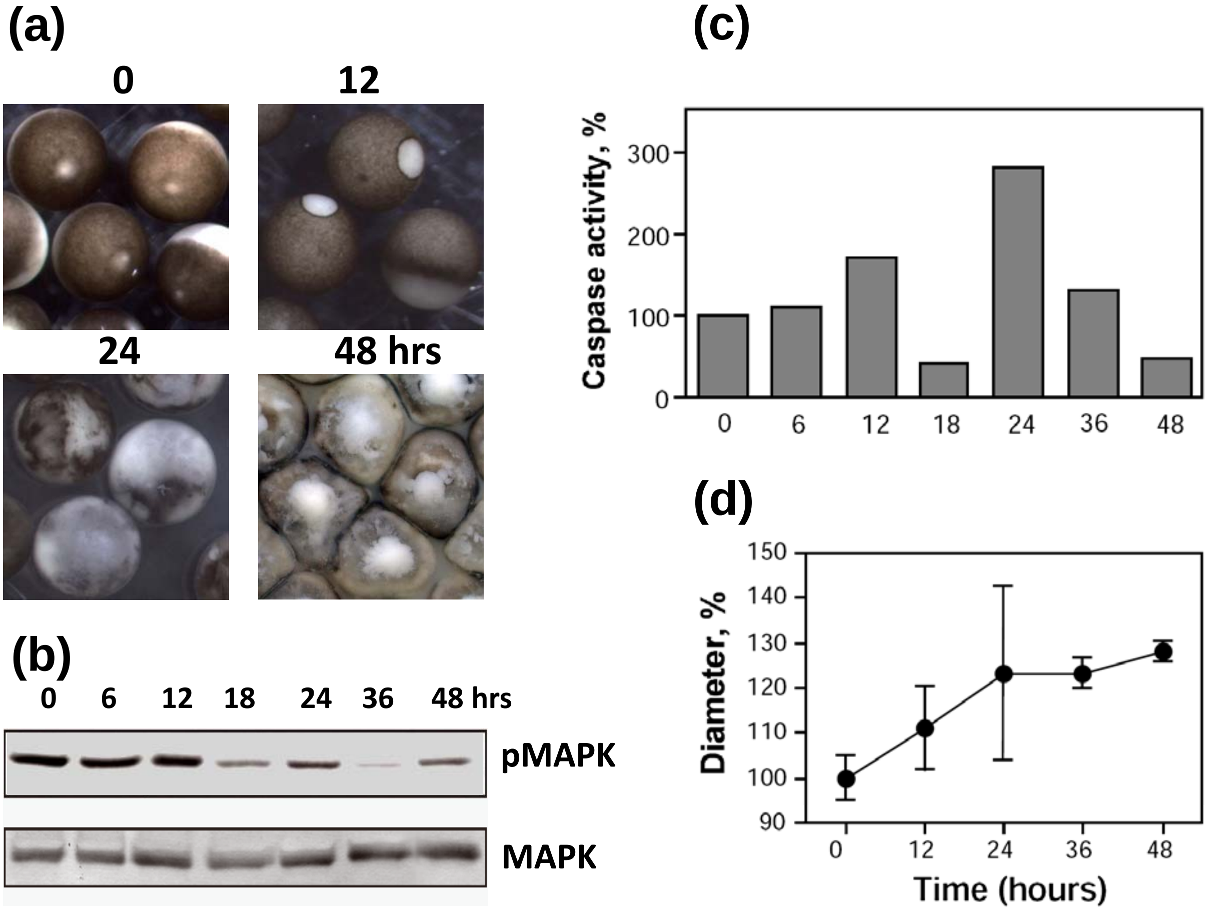

Figure S3. Degradation of unfertilized dejellied *Xenopus* eggs deposited into DB buffer. **(a)** Changes in egg morphology, **(b)** MAPK dephosphorylation, **(c)** caspase activation, and **(d)** egg diameter. Data in panel (d) were obtained by measuring five eggs.
