# Supplementary material for: Unfertilized frog eggs die by apoptosis following meiotic exit
Source: BMC Cell Biol. 2011 Dec 23;12:56. doi: 10.1186/1471-2121-12-56 (PMC3268744; doi:10.1186/1471-2121-12-56)
Supplement: Additional file 4 — Figure S4. Apoptotic degradation of roscovitine-treated Xenopus eggs. Freshly squeezed dejellied eggs were placed into OR-2 buffer and treated with 50 μM roscovitine. Egg morphology (a), Mos, cyclin B2 levels and MAPK activation state (b), caspase 3 activity (c), intracellular ATP content (d), ADP/ATP ratio (e), and egg diameter (f) have been monitored at the indicated times. Bars in panel (f) represent SD of the mean obtained by measurement of seven eggs. [file 1471-2121-12-56-S4.PDF]

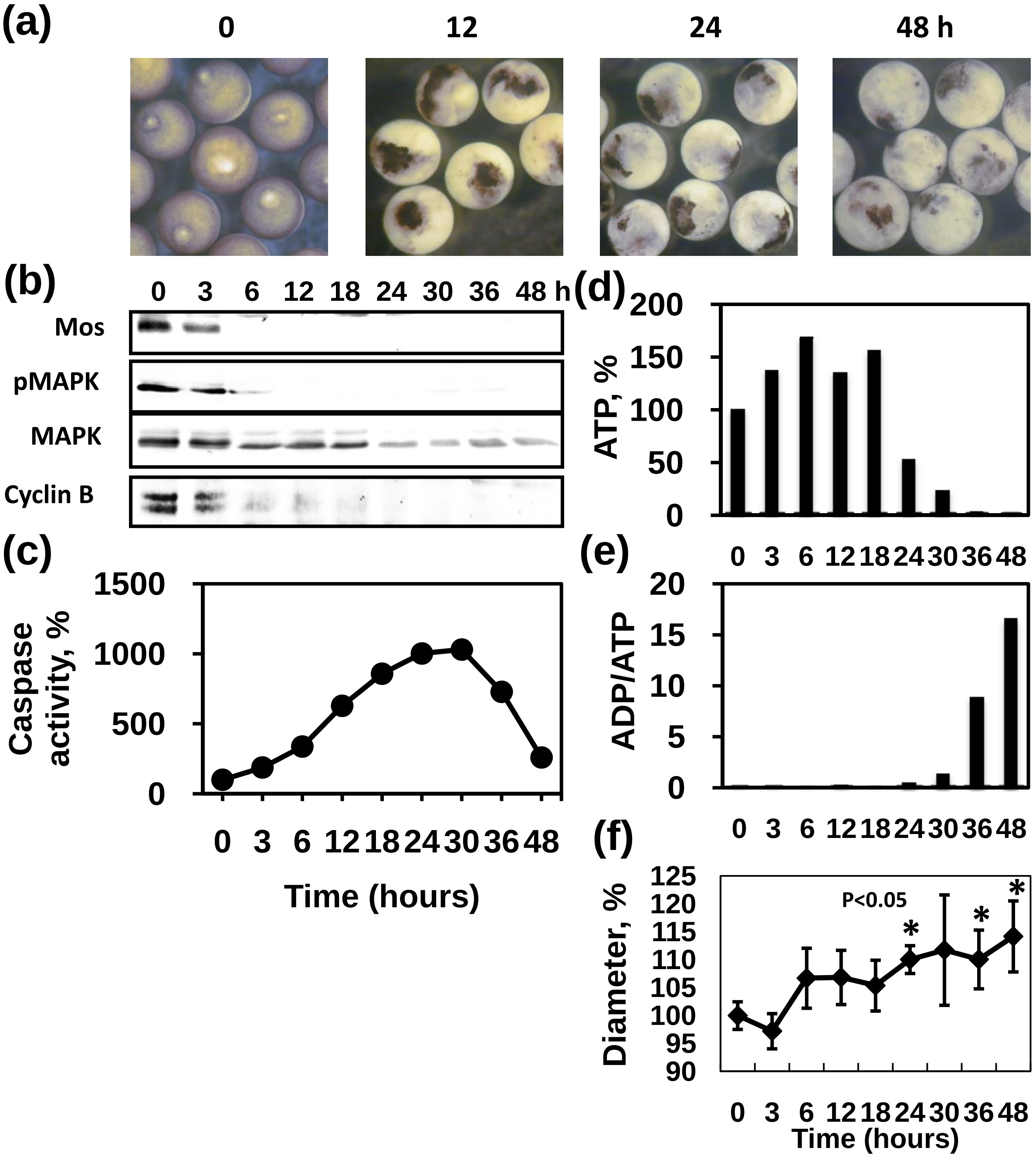

Figure S4. Apoptotic degradation of roscovitine-treated *Xenopus* eggs. Freshly squeezed dejellied eggs were deposited into OR-2 buffer and treated with 50 µM roscovitine. Egg morphology **(a)**, Mos, Cyclin B2 levels and MAPK activation state **(b)**, caspase 3 activity **(c)**, intracellular ATP content **(d)**, ADP/ATP ratio **(e)**, and egg diameter **(f)** have been monitored at the indicated times. Bars in panel **(f)** represent SD of the mean obtained by measurement of seven eggs.
